# Eating Disorders and Alcohol Use Disorders

**Published:** 2002

**Authors:** Carlos M. Grilo, Rajita Sinha, Stephanie S. O’Malley

**Affiliations:** Carlos M. Grilo, Ph.D., is an associate professor in the Department of Psychiatry and director of the Eating Disorder Program; Rajita Sinha, Ph.D., is an associate professor in the Department of Psychiatry and director of the Substance Abuse Treatment Unit; and Stephanie S. O’Malley, Ph.D., is a professor in the Department of Psychiatry and director of the Division of Substance Abuse Research, all at Yale University School of Medicine, New Haven, Connecticut

**Keywords:** eating disorder, personality disorder, AODD (alcohol and other drug dependence), comorbidity, anorexia nervosa, bulimia nervosa, gender differences, diet, coping skills, cognitive therapy, behavior therapy, psychosocial treatment method

## Abstract

Alcoholism and eating disorders frequently co-occur and often co-occur in the presence of other psychiatric and personality disorders. Although this co-occurrence suggests the possibility of common or shared factors in the etiology of these two problems, research to date has not established such links. Regardless of the precise meaning of the association, the reality that eating disorders and alcohol use disorders frequently co-occur has important implications for assessment, treatment, and future research.

Numerous studies suggest that eating disorders (EDs) and alcohol and other drug use disorders (referred to throughout this paper as substance use disorders [SUDs]) frequently co-occur and often co-occur in the presence of other psychiatric and personality disorders. This review will consider the extent and nature of such co-occurrences and whether research supports the possibility of common or shared factors in the etiology or maintenance of EDs and SUDs. The reality that EDs and SUDs frequently co-occur has important implications for assessment, treatment, and future research. Although this review will offer implications for clinicians and researchers in *both* fields, the presentation bias will be toward providing a more detailed discussion of the ED literature for professionals in the alcoholism field.

## Eating Disorders

The current classification system, the fourth edition of the *Diagnostic and Statistical Manual of Mental Disorders* (DSM–IV) (American Psychiatric Association [APA] 1994) specifies three ED diagnoses. The formal diagnoses are anorexia nervosa (AN), bulimia nervosa (BN), and eating disorder not otherwise specified (EDNOS). In addition, the DSM–IV includes a new ED category (binge eating disorder [BED]) as a research category. BED is a specific example of EDNOS. Brief descriptions of these EDs follow. See Grilo (2002*a*) and Becker and colleagues (1999) for detailed discussions of the clinical features and assessment issues of these disorders.

***Anorexia Nervosa*** is characterized by a refusal to maintain normal body weight (defined as 15 percent below normal weight for age and height), an intense fear of becoming fat, and (in females) skipped menstrual periods (i.e., amenorrhea) for at least 3 months. People with AN have a severely distorted body image. They see themselves as overweight despite being overly thin, and they tend to deny the seriousness of their low body weight. The DSM–IV specifies two subtypes of AN—a “restricting type,” characterized by strict dieting or exercise without binge eating; and a “binge-eating/purging type,” marked by episodes of binge eating and/or purging via self-induced vomiting or misusing laxatives, enemas, or diuretics. In severe cases, medical complications or death from starvation can occur. Roughly 50 percent of people with AN may eventually develop bulimia nervosa (described below). AN is a rare disorder; it occurs disproportionately in women, and is estimated to have a prevalence of roughly 1 percent in adolescent and young adult women (Hoek 1993).

***Bulimia Nervosa*** is characterized by recurrent episodes of binge eating (defined as consuming unusually large amounts of food in a discrete period of time plus a subjective sense of lack of control over eating). BN is further characterized by regular use of extreme weight control methods (e.g., vomiting; abuse of laxatives, diet pills, or diuretics; severe dieting or fasting; vigorous exercise) and by dysfunctional attitudes about weight or shape that unduly influence self-evaluation. For a diagnosis of bulimia nervosa, the DSM–IV requires both the binge eating and inappropriate weight control methods to have occurred, on average, at least twice per week during the past 3 months. The DSM–IV specifies two subtypes of BN, a “purging type” and a “non-purging type,” which is limited to the severe dieting, fasting, or exercise forms of weight control behaviors. If either form of BN occurs during a current episode of AN, the assigned diagnosis is AN. BN, like AN, is more common in females and has an estimated prevalence rate of 2 percent to 3 percent in young females (Kendler et al. 1991; Kringlen et al. 2001).

***Eating Disorder Not Otherwise Specified*** is generally considered the most prevalent form or category of ED and the least studied (Andersen et al. 2001; Grilo et al. 1997). Outside of research centers with specific recruitment requirements, the majority of patients who present for treatment for eating-related problems are “partial syndrome” or EDNOS cases. That is, they fail to meet all the diagnostic requirements for one of the “formal” EDs but they have significant symptoms and associated problems. Indeed, researchers have questioned the significance of the failure to meet some of the specific criteria (such as the necessity that amenorrhea be present in female patients for a diagnosis of AN to be made). Investigators have also claimed that some of the diagnostic criteria for the formal EDs are too stringent (Andersen et al. 2001; Striegel-Moore et al. 2000). Many patients experience significant and clinically meaningful problems with eating and body image, but do not always fulfill the exact requirements for the diagnoses of AN or BN.

***Binge Eating Disorder****, *included as a provisional category in the DSM–IV, is a specific example of an EDNOS. BED is characterized by recurrent episodes of binge eating (an average of 2 days with binge episodes per week over a 6-month period is required; marked distress exists because of the binge eating) without the compensatory weight control methods that are required for the diagnosis of BN (Grilo 1998, 2002*a*). BED, unlike AN and BN, is not uncommon in males or in people of color. It is most frequently seen in adults, and has an estimated prevalence of 3 percent in adults and roughly 8 percent in obese persons (Spitzer et al. 1993). BED is associated with increased risk for obesity and thus for the plethora of medical problems associated with obesity (Grilo 1998).

### Age Ranges and Gender

Eating disorders most frequently develop during adolescence or early adulthood, but their onset can occur during childhood or much later in adulthood (Grilo 2002*a*). The peak age range for onset of AN is 14 to 18 years, although some patients develop AN as late as their 40s (Frey 1999). Similarly, the peak age range for BN is adolescence through early adulthood (Lamb 1999). BED most frequently occurs in young to middle adulthood (Grilo 1998). Although developmental challenges and severe dieting generally predate AN and BN, it appears that a significant proportion of people with BED report no dieting prior to the onset of binge eating (Grilo 1998, 2002*a*). Although AN and BN occur mostly in females and BED is more common in females than males, it is important to not overlook these EDs in men (Andersen 1995; Andersen and Holman 1997). Available research suggests that among ED patients, few gender differences exist in the specific features of the EDs (Barry et al. 2002; Woodside et al. 2001).

## Co-Occurrence of Substance Use Disorders and Eating Disorders

Most studies have reported that EDs and SUDs frequently co-occur, with especially high rates observed among patients in treatment (e.g., Beary et al. 1986; Brewerton et al. 1995; Bushnell et al. 1994; Goldbloom et al. 1992; Grilo et al. 1995*b*; Higuchi et al. 1993; Suzuki et al. 1993; Taylor et al. 1993; see reviews by Grilo et al. 1995*a* and Holderness et al. 1994 for more complete listings of earlier studies).

Although research has generally reported high rates of co-occurrence between EDs and SUDs, perhaps most striking is the marked inconsistency or variability in the reported co-occurrence rates across studies. A previous review (Holderness et al. 1994) noted that estimates of BN in patients with SUDs ranged from 8 percent to 41 percent and estimates for AN ranged from 2 percent to 10 percent. Methodological issues account, in part, for some of the inconsistencies in the reported co-occurrence rates and make interpretation of the literature ambiguous. Variations in recruitment methods (community versus treatment samples, and, if using treatment samples, the type of treatment facility [e.g., general psychiatric, substance or chemical dependency, eating disorder; inpatient versus outpatient]) and assessment and diagnostic methods (survey, self-report, diagnostic interview) account for some of the variability in the literature.

These important methodological limitations notwithstanding, research does suggest that EDs and SUDs frequently co-occur. Therefore, research seeks to determine the significance of the co-occurrence for informing (a) models of etiology and pathophysiology, and (b) approaches to treatment and clinical management. The remainder of this article will review this research, first examining the prevalence of the co-occurrence of EDs and SUDs in more detail and then exploring whether research supports the possibility of common or shared factors in the etiology or maintenance of EDs and SUDs.

### Co-Occurrence versus Comorbidity

“Comorbidity” is a widely used term in psychopathology research but one that appears to reflect various meanings or definitions. Kendall and Clarkin (1992), among others (Grilo 2002*b*), noted numerous possible meanings of comorbidity, including random co-occurrence of disorders that are independent, co-occurrence of different disorders that share a common etiology, or different disorders that have a causal relationship between them. Comorbidity may reflect, in part, artifacts of the diagnostic systems because of criterion overlap (e.g., one criterion for borderline personality disorder is “impulsivity,” which can be met in part by binge eating and/or by substance use). As demonstrated initially by Berkson (1946), and more recently by duFort and colleagues (1993), studies of treatment-seeking patients must be interpreted cautiously because of biases (i.e., people seeking treatment may have especially severe problems, people with multiple problems may seek treatment for one or several of the problems) that can make interpretation of comorbidity difficult and may limit generalizability to community samples. Thus, a starting point for possible comorbidity is when rates of co-occurring diagnoses are statistically different from those expected, given the base rates for the individual disorders (Kraemer 1995). Allison (1993) maintained the importance of selecting “relevant” control or comparison groups to provide a context for interpreting differences in co-occurrence patterns. Appropriate comparisons might include psychiatric patient groups without the eating and/or alcohol use disorders.

Recent research with both people in treatment (Grilo et al. 1995*b*; Wilfley et al. 2000) and in the general population (Dansky et al. 2000; von Ranson et al. 2002; Telch and Stice 1998; Yanovski et al. 1993) that used systematic recruitment methods, standardized diagnostic interviews (rather than self-report), and “relevant” comparison groups has revealed that, although EDs and SUDs co-occur, the co-occurrence is either not significantly greater or—if so—is only marginally greater than the co-occurrence rate in relevant comparison groups (i.e., patient groups of comparable severity chosen to provide a context as opposed to the frequently used “normal” control group). Grilo and colleagues (1995*a*, 1995*b*) found that, although EDs are frequently diagnosed among inpatients with SUDs, they are also frequently diagnosed in other psychiatric inpatients. In this controlled study, the frequency of AN and BN was not greater in patients with SUDs than without SUDs. Subthreshold manifestations of EDs (i.e., EDNOS; cases where insufficient criteria were present to warrant either BN or AN diagnoses) were diagnosed significantly more frequently in the patients with SUDs than without. Some research has also suggested that patients with nonpurging AN may be *less* likely than patients with other forms of EDs, including AN purging subtype, to have SUDs. The increased possibility for SUDs to co-occur with atypical manifestations of EDs, rather than with AN and BN, is examined further in the following section.

The three studies of comorbidity in BED that used relevant comparison groups found high rates of lifetime alcohol use and SUDs but not higher rates than observed in the comparison groups (Telch and Stice 1998; Wilfley et al. 2000; Yanovski et al. 1993). Most recently, von Ranson and colleagues (2002) reported findings from a large community study of two groups (672 adolescent girls, 718 adult women) assessed using diagnostic interviews. The authors reported that EDs and substance use were positively related, but the association was not significant. They concluded that there is no strong overarching relationship between these problems. These findings suggest caution in interpreting comorbidity between different forms of EDs and SUDs.

### Other Comorbid Psychiatric Disorders

Although this review focuses primarily on the co-occurrence of EDs and SUDs, both of these classes of disorders frequently co-occur with other forms of psychopathology. A large body of research has documented associations between EDs and other psychiatric and personality disorders (Bulik et al. 1997; Grilo 2002*b*; Grilo et al. 1995*a*, *b*) as well as between SUDs and other psychiatric disorders (Grilo et al. 1997; Sher and Trull 2002).

Controlled studies (Dansky et al. 2000; Grilo et al. 1995*a*, *b*; Wiseman et al. 1999) have suggested that some of the apparent co-occurrence between EDs and SUDs may be related, in part, to other psychiatric comorbidities. Specifically, Dansky and colleagues (2000) reported that the relationship between BN and alcohol use disorders reported by the National Women’s Study was likely indirect and the result of associations with other psychiatric disorders, most notably major depressive disorder and post-traumatic stress disorder. Grilo and colleagues (1995*b*) compared inpatients who had ED with and without SUD with a comparison group who had SUD but not ED. In this controlled comparison, personality disorders characterized as cluster B (i.e., erratic or unstable) were diagnosed more frequently in the patients with co-occurring ED and SUD, whereas cluster C personality disorders (i.e., anxious or fearful) were diagnosed more frequently in patients with ED without co-occurring SUD. This three-group comparison allowed for a finer distinction regarding potential comorbidity and raised the possibility of subgroups of patients (e.g., with borderline personality disorder) who might be most likely to have problems with both eating and substance use disorders. Consistent with this, Bulik and colleagues (1997) found that, although women with alcoholism and BN had higher rates of a variety of psychiatric problems than women with BN without histories of alcohol use disorders, multivariate analyses revealed that borderline personality disorder was the sole distinguishing variable between the two groups. Most recently, Wiseman and colleagues (1999) found that the order of onset of the two disorders might be important. Patients who developed EDs early and prior to SUDs had greater levels of psychiatric and personality disorder psychopathology compared with patients who developed the ED after the SUD and with patients who had an ED but no SUD.

These findings suggest that additional psychiatric disorders frequently co-occur with EDs and SUDs, and may play a role in their relationship to each other. In particular, these findings suggest that patients who suffer from both eating disorders and substance abuse disorders may have deficits in impulse control. Related to this line of investigation, recent years have witnessed increased attention to the potential role of childhood abuse, perhaps mediated by personality disorders, as a common factor in patients with both EDs and SUDs. Research, however, has not generally supported specific or strong associations between childhood abuse and specific disorders (Grilo and Masheb 2001; Smolak and Murnen 2002). Another issue to examine in the relationship between these disorders is the significant frequency with which ED symptoms occur with SUDs.

### Eating Disorder Symptoms Among Women with Substance Use Disorders

Grilo and colleagues (1995*a*) have reported that EDNOS (but not AN or BN) was significantly more common in people with SUD than without SUD. This suggests that it is important for clinicians to consider and screen for subthreshold levels of EDs in addition to formal ED diagnoses. Moreover, assessment of co-occurring subthreshold eating problems may facilitate earlier intervention to prevent later development of the full-blown disorder.

A few studies have examined the specific features of EDs present among patients with SUDs (Sinha et al. 1996; Peveler and Fairburn 1990; Jackson and Grilo in press). Sinha and colleagues (1996) assessed eating behaviors and the attitudinal features of EDs in a community-based sample of 201 young women (ages 18 to 30) who comprised the following four groups: alcohol dependent, alcohol dependent with anxiety disorders, anxiety disorders only, and neither alcohol nor anxiety disorders. Women with alcohol dependence had significantly higher levels of the behavioral and attitudinal features of eating disorders and were more likely to meet the criteria for BN and EDNOS than women without alcohol dependence. Interestingly, these authors found that alcoholism was more closely related to the attitudinal features, whereas anxiety disorders were more closely associated with the behavioral features of eating disorders.

### Eating Disorder Symptoms by Gender and Ethnicity

More recently, Jackson and Grilo (in press) examined the specific features of EDs and tested for gender and ethnic differences in a racially diverse group of outpatients with SUDs. Similar to previous studies with primarily Caucasian samples (Peveler and Fairburn 1990; Sinha et al. 1996), eating-related problems were not uncommon in substance abusers. Roughly 20 percent of men and women reported binge eating, and 12 percent reported some form of inappropriate weight compensatory behaviors. Problematic attitudes about body shape were also common; 28 percent of the Jackson and Grilo (in press) sample reported overvalued ideas regarding shape at levels considered to be clinically significant—as compared with 28 percent in the study of young women reported by Sinha and colleagues (1996) and 26 percent in the study reported by Peveler and Fairburn (1990). Jackson and Grilo (in press) found no significant ethnic differences in obesity, in features of eating disorders, or in levels of body image dissatisfaction. Men and women were similar in terms of overweight and behavioral features, but women had significantly higher levels of attitudinal features of EDs. Thus, contrary to clinical lore, weight- and eating-related problems are not uncommon in males or in minority groups.

## Research Investigating Whether Common Factors May Underlie the Co-Occurrence of EDs and SUDs

The studies described above demonstrate that EDs and SUDs often co-occur and that ED symptoms are significantly more common in people with SUDs than without SUDs. Although research is ongoing, reasons for this co-occurrence have not been reported. One potential explanation is that these disorders are different manifestations of a common underlying factor. Three types of research provide support for this hypothesis: studies of dieting and substance use, studies of brain chemistry, and family and genetic studies.

### Studies of Dieting Behavior and Substance Use

Research has documented significant associations between dieting and eating problems and substance use in younger populations. Krahn and colleagues (1992), for example, found that among college women, increasing severity of dieting and problems associated with EDs were associated with increased rates of alcohol, cigarette, and other drug use. Krahn and colleagues (1996) also found that dieting during pre-adolescence (among sixth grade students) predicted future alcohol use. Such findings, when considered with studies showing that food deprivation can increase self-administration of alcohol and other drugs in laboratory animals, are consistent with models positing that common mechanisms may play a role in EDs and SUDs (see Krahn 1991). For example, Krahn (1991) suggested that food deprivation might cause alterations in the central nervous system’s reward pathways, thus increasing the consumption of reinforcing substances (e.g., alcohol). However, as emphasized above, and by other reviews (Wilson 1993), the fact that these problems are associated does not demonstrate a specific or common cause.

### Studies of Brain Chemistry

Animal studies of brain chemistry have provided some support for the view that EDs and alcohol use disorders may have some shared factors. Some research, for example, has suggested that both disorders may be related to atypical endogenous opioid peptide (EOP) activity. EOPs have been found to influence both alcohol and food consumption (see Mercer and Holder 1997) and may play roles in the control of eating behavior (Berridge 1996; Carr 1996; Cooper and Kirkham 1993; Gosnell and Levine 1996) as well as the development of alcoholism (Reid 1985; Reid et al. 1991; see also Froehlich 1995). In addition, brain neurotransmitter systems, including the serotonin, gamma-aminobutyric acid (GABA), and dopamine systems, are the focus of active research across a wide range of psychiatric and behavioral problems, including food and alcohol consumption (see Mercer and Holder 1997). Particularly active attention has been paid to the role of serotonin, which has been implicated in the control of eating, mood, and impulsivity (Brewerton 1995; Kaye et al. 1998). In addition, treatment studies have reported some support for the efficacy of selective serotonin reuptake inhibitors (SSRIs) across different EDs (Fluoxetine Bulimia Nervosa Collaborative Study Group [FBNCSG] 1992; Hudson et al. 1998; Kaye et al. 2001).

### Family and Genetic Studies

Early research reported that people with eating disorders are more likely than those without EDs to have family histories of substance use disorders (e.g., Hudson et al. 1983; Jones et al. 1985). However, several recent large, carefully conducted studies have found that EDs (especially BN) and SUDs segregate *independently* in families—that is, eating disorders and substance use disorders most likely do not have the same genetic, familial, and environmental risk factors. For example, Kaye and colleagues (1996) reported that alcohol or other drug dependence was increased only in first-degree relatives of women with BN who themselves also had alcohol or other drug dependence. Schuckit and colleagues (1996), in a large study of alcohol-dependent people and their relatives, also reported weak evidence at best for familial transmission between alcohol dependence and BN. Lilenfeld and colleagues (1997) reported that women with co-occurring BN and SUD have higher rates of problems with anxiety, a variety of personality disturbances including antisocial behavior, and high rates of familial SUD, anxiety, impulsivity, and affective instability. These authors hypothesized that a familial vulnerability for impulsivity and affective instability may contribute to the development of SUD in a subgroup of BN patients. Using data from a large epidemiological sample of female twin pairs, Kendler and colleagues (1995) demonstrated that most of the genetic factors associated with vulnerability to alcoholism in women do not alter the risk for development of BN.

## Treatment of Co-Occurring Alcoholism and Eating Disorders

Although alcoholism and other SUDs frequently occur with EDs, research has not established common or shared factors in the etiology or maintenance of this co-occurrence. Nonetheless, the frequent co-occurrence of problems with eating and alcohol may signal greater psychiatric disturbances (Grilo et al. 1995*b*) and greater medical risk (Catterson et al. 1997; Mitchell et al. 1991). These clinical realities represent considerable challenges to practitioners and researchers. The most common questions include how to identify the presence of possible problems, which problem to focus on first, or whether/how to address both concurrently (Daniels et al. 1999; Wilson 1993; Mitchell et al. 1997). These are important questions and there is a pressing need for research on these treatment issues (Grilo et al. 1997). Not only has little research been done on treating these co-occurring conditions, but many treatment studies with ED patients either exclude patients with substance dependence or enroll few such patients. Although the brief overview that follows will offer implications for clinicians and researchers in both fields, this section gives a more detailed discussion of the ED intervention literature for professionals in the alcoholism field.

### Assessment and Screening for Eating Disorders

Good, comprehensive assessment of patients is necessary for good treatment. Assessment protocols should involve questionnaires (i.e., instruments) that are sensitive enough to flag patients with potential problems for further evaluation. Failure to identify all problems may contribute to poor retention and treatment outcomes even for the targeted problem. Screening instruments for alcohol problems are described in detail elsewhere (Bradley et al. 1998). Although standardized interviews are generally thought to hold important advantages for accurate and thorough assessment of EDs (Grilo et al. 2001*a*), it may not be possible or practical for many types of clinical facilities to use them because of cost, time, and lack of training.

The authors of this article recommend two self-report instruments for the screening and preliminary assessment of EDs. The first is the *Questionnaire on Eating and Weight Patterns–Revised* (QEWP–R) (Yanovski et al. 1993), a well-established and easy-to-complete self-report instrument. The QEWP–R, widely used in research programs, screens for the presence of the specific ED categories and provides useful information about the frequency of problem eating and dieting behaviors. The second instrument is the *Eating Disorder Examination–Questionnaire Version* (EDEQ) (Fairburn and Beglin 1994), the self-report version of the *Eating Disorder Examination* interview (Cooper and Fairburn 1987). The EDEQ offers a number of advantages over other self-report measures and provides detailed information about the behavioral and attitudinal features of eating disorders. The EDEQ has received some support for its utility (Grilo et al. 2001*a*) and has been used with substance abusers (Black and Wilson 1996). The relative merits of different assessment methods are described elsewhere (Grilo et al. 2001*a*). Briefly, such instruments are generally thought to underestimate the frequency of some of the behavioral features of EDs (e.g., binge eating) and overestimate some of the cognitive or attitudinal symptoms, compared with interviews (Grilo et al. 2001*a*). These limitations notwithstanding, such screens are useful for efficiently identifying people with possible problems. Of course, in addition, it is important for clinicians and researchers alike to consider comprehensive medical and psychiatric evaluations for these patient groups (see Grilo 1998). In particular, patients with these co-occurring problems require careful medical evaluation and followup (Mitchell et al. 1991). In terms of followup, it may be particularly useful for repeated assessments to include the ED screens. Some clinical experience suggests the possibility that successful cessation of substance or alcohol use may be followed by the re-emergence of ED symptoms in some patients. Although this hypothesis awaits conclusive research, it highlights the usefulness of repeated assessments.

### Pharmacological Treatments

Pharmacological treatments have generally been found to have little effect on AN either as the primary approach or as an augmentation approach (Attia et al. 1998), although the antidepressant fluoxetine was found to decrease frequency of relapse in one study (Kaye et al. 2001). In contrast, pharmacological treatments, particularly antidepressant medications, have generally been found to be superior to placebo for the treatment of BN (e.g., Agras et al. 1992; FBNCSG 1992; Mitchell et al. 1990) and BED (e.g., Hudson et al. 1998; McCann and Agras 1990; McElroy et al. 2000; see Grilo 1998). It is worth stressing that these studies generally find, particularly for fluoxetine, that high doses are required to produce effects (as high as 60 mg per day in the case of fluoxetine) (FBNCSG 1992). Unfortunately, surveys have revealed that most patients with BN treated with pharmacotherapy by community practitioners received inadequate dosing (Crow et al. 1999). Nevertheless, fluoxetine has also been shown to reduce depressive symptoms and alcohol consumption in depressed alcoholics (Cornelius et al. 1997). Controlled research testing the efficacy of this medication among women with both alcoholism and EDs is needed.

Medications designed to block the action of opioids (i.e., opioid antagonists) have demonstrated efficacy for reducing alcohol use and relapse, and increasing abstinence rates among alcoholic patients (Anton et al. 1999; Heinala et al. 2001; Mason et al. 1999; Monti et al. 2001; O’Malley et al. 1992; Volpicelli et al. 1992, 1997; see also Krystal et al. 2001). The opioid antagonist naltrexone (ReVia™) has also been studied as a treatment for ED. One study that compared naltrexone, imipramine, and placebo among BED patients found that both medications produced reductions in binge eating but neither was superior to placebo (high placebo response occurred in this study) (Alger et al. 1991). One study found that naltrexone reduced the frequency of binge eating in patients with BN during the first few weeks of treatment but that the effects did not last (Jonas and Gold 1987). A rigorous controlled study is currently under way at Yale University to evaluate the efficacy of naltrexone among alcoholic women and women with both alcoholism and EDs.

### Psychological Treatments of Eating Disorders

Cognitive behavioral therapy (CBT) has received the most consistent support of any psychological or pharmacologic treatment for EDs. Briefly, CBT is a focal and structured treatment that involves a collaborative effort between patients and clinicians (Fairburn et al. 1993*a*). CBT for eating disorders can be delivered via individual or group approaches and generally follows three phases. The first phase involves education and presentation of the treatment model, including expectations for treatment and homework, teaching behavioral strategies such as self-monitoring to identify problems, and a graded approach to normalization of eating. The second phase involves the use of cognitive restructuring methods to identify, challenge, and modify maladaptive thinking. The final stage involves relapse prevention techniques and problem solving to generalize the skills to other areas and to consolidate improvements. CBT has been found to be superior to control conditions, to most other forms of psychological therapies, to behavioral therapies without the cognitive components, and to the pharmacological treatments (e.g., Agras et al. 1992, 2000; Fairburn et al. 1993*a*; see reviews: Wilson and Fairburn 1998; Grilo 1998, 2000). Moreover, self-help versions (e.g., Fairburn 1995) of standard CBT therapist manuals (Fairburn et al. 1993*b*) have demonstrated efficacy (Carter and Fairburn 1998; Peterson et al. 1998; Treasure et al. 1996). This approach may provide general practitioners with expertise in CBT with the technology to help certain ED patients.

Although CBT is generally regarded as the first-line treatment of choice for ED (Agras et al. 2000; Wilson and Fairburn 1998), research is needed to determine its usefulness for patients with co-occurring alcoholism and eating disorders, and to develop integrated psychological treatment approaches for patients with alcoholism and eating disorders (Mitchell et al. 1997). Although the data are sparse, the treatment literature has not suggested that alcoholism or a history of alcoholism diminishes CBT treatment effectiveness for BN or BED (Goldbloom 1993; Mitchell et al. 1990; Wilfley et al. 2000). No available studies have examined whether eating disturbances influence the outcome of alcoholism treatment. Although clinical lore suggests that personality disorders––if present––are associated with negative treatment outcomes, this has not received empirical support in treatment studies of patients with EDs (Grilo 2002*b*), and findings from treatment studies of patients with SUD are mixed (Grilo and McGlashan 1999).

Based on clinical experience with both patient groups, the authors suggest that certain CBT-based treatments represent a good starting point for treating co-occurring alcohol use and eating disorders. Basic aspects of the cognitive behavioral approach (e.g., coping skills therapy) have been found effective for treating alcohol dependence (Kadden et al. 1992; Monti et al. 1989, 2001) and are useful for ED patients. However, as previously noted, behavioral therapies without the specific cognitive components of CBT have inferior long-term outcomes compared with CBT (Fairburn et al. 1993*a*). Nevertheless, specific forms of behavioral and coping skills treatments (without the specific cognitive components of the CBT approaches for EDs) have been used successfully with substance abusers and seem to be readily integrated with pharmacological approaches (Monti et al. 2001; O’Malley et al. 1992; Sinha 2000).

Thus, an approach that targets alcohol use and pathologic eating behaviors may be especially appropriate for treating patients with both disorders. Treatment designed to teach new coping skills to patients with alcoholism could also have a beneficial effect on eating disorders even if the ED is not specifically targeted. Given the well-known ambivalence that characterizes many of these patients (e.g., Vitousek et al. 1998), another potentially relevant approach involves motivational enhancement interviewing (Rollnick and Miller 1995), which has received some support for SUDs (Project MATCH Research Group 1997) and EDs (Treasure et al. 1999).

Another promising approach is dialectical behavior therapy (DBT), which initial research supports for both BN (Safer et al. 2001) and BED (Telch et al. 2001). DBT, which focuses on awareness of problems and choices, mood regulation techniques, and coping skills, directly addresses many of the needs of both ED and alcohol use disorder patients, including the frequently co-occurring borderline personality disorder. Indeed, the initial treatment outcome findings for DBT for both BN (Safer et al. 2001) and BED (Telch et al. 2001) suggest that addressing a potential vulnerability (e.g., problems with mood regulation and coping) can lead to improvements in ED even without a direct focus on the eating behaviors, a finding that parallels that reported for interpersonal psychotherapy (Agras et al. 2000; Fairburn et al. 1993*a*; Wilfley et al. 1993). Telch and colleagues (2001) speculated that DBT may be particularly helpful for ED patients characterized by high levels of negative affect. Recent studies with BN (Grilo et al. 2001*b*; Stice and Agras 1999) and BED (Grilo et al. 2001*c*) revealed two subtypes of these EDs: dietary and a mixed dietary–negative affect. The dietary subgroup was characterized primarily by eating-specific psychopathology without associated problems with self-esteem and depression (negative affect). Patients with the mixed dietary-negative affect subtype also had high rates of alcohol and other drug problems. It is possible that such patients, particularly if they have problems with impulsivity or have co-occurring borderline personality disorder, might benefit from affect regulation and coping skills approaches such as DBT.

## Conclusion

Alcoholism and EDs frequently co-occur and often co-occur in the presence of other psychiatric and personality disorders. Although such diagnostic co-occurrence suggests the possibility of shared factors in the etiology or maintenance of these problems, research has not established such links. The clinical reality that eating and alcohol use disorders frequently co-occur has important implications for assessment, treatment, and research. Comprehensive assessment is necessary for good treatment. Research on methods of treating people with co-occurring alcohol and eating problems represents a major need. Until further guidance is provided, the authors recommend concurrently addressing both disorders. CBT, coping skills, or DBT approaches seem to be reasonable starting points.
